# Supplementary material for: Conformational Stabilization of Gp41-Mimetic Miniproteins Opens Up New Ways of Inhibiting HIV-1 Fusion
Source: Int J Mol Sci. 2022 Mar 3;23(5):2794. doi: 10.3390/ijms23052794 (PMC8911282; doi:10.3390/ijms23052794)
Supplement: Supplementary file 1 [file ijms-23-02794-s001.zip › ijms-1603900-supplementary.pdf]

International Journal of *Molecular Sciences*

MDPI

Supplementary information to:

Conformational stabilization of gp41-mimetic miniproteins opens up new ways of inhibiting HIV-1 fusion

Mario Cano-Muñoz[1,2,*], Julie Lucas[2], Lin Li-Yun[2], Samuele Cesaro[1,†], Christiane Moog[2] and Francisco Conejero-Lara[1,*]

[1] Departamento de Química Física, Instituto de Biotecnología y Unidad de Excelencia de Química Aplicada a Biomedicina y Medioambiente (UEQ), Facultad de Ciencias, Universidad de Granada, 18071 Granada, Spain; mariocano@ugr.es, conejero@ugr.es

[2] INSERM U1109, Fédération de Médecine Translationnelle de Strasbourg (FMTS), Université de Strasbourg, 67084 Strasbourg, France; julie.lucas@etu.unistra.fr (J.L.); li-yun.lin@etu.unistra.fr (L.L.-Y.); c.moog@unistra.fr (C.M.)

* Correspondence: mariocano@ugr.es (M.C.-M.); conejero@ugr.es (F.C.-L.); Tel.: +34-958242371 (F.C.-L.)

† Present address: Department of Neurosciences, Biomedicine and Movement Sciences, Section of Biological Chemistry, University of Verona, Strada Le Grazie 8, 37134 Verona, Italy.

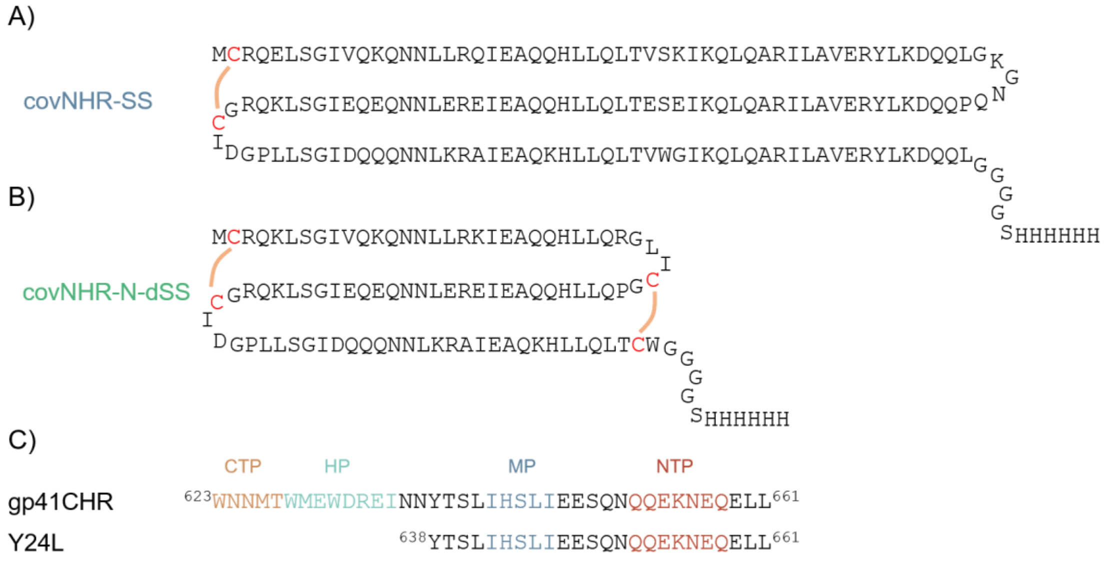

Figure S1. Sequences and topology of the covNHR miniproteins and peptides. Mutations in red were engineered in this work. A) covNHR-SS sequence. B) covNHR-N-dSS sequence C) CHR C34 and Y24L peptide sequences. Residues corresponding to the different binding pocket motifs are colored as follows: CTP in orange, HP in cyan, MP in blue and NTP in red.

Table S1. Description of envelope glycoproteins used in the ELISA binding experiments.

Abbreviation	Type	Description	Reference
JRFL	Gp140	A soluble uncleaved gp140 Env stabilized trimer derived from HIV-1 JRFL containing a C-terminal foldon sequence.	[1]
MN/LAI	Gp160	A hybrid oligomeric gp160 Env with gp120 derived from HIV-1 MN and gp41 derived from HIV-1 LAI.	[2]
THO23/LAI	Gp160	Recombinant Env with gp120 from HIV-1 92TH023 linked to gp41 from LAI, with a deletion in the immunodominant region.	[3]
ZM 4096	Gp140	Synthetic construct derived from gp140 sequence of the codon-optimized HIV-1 96ZM651.	[4]
CN54	Gp140	Recombinant Env trimer containing gp120+gp41 ectodomain from HIV-1 CN54.	[5]

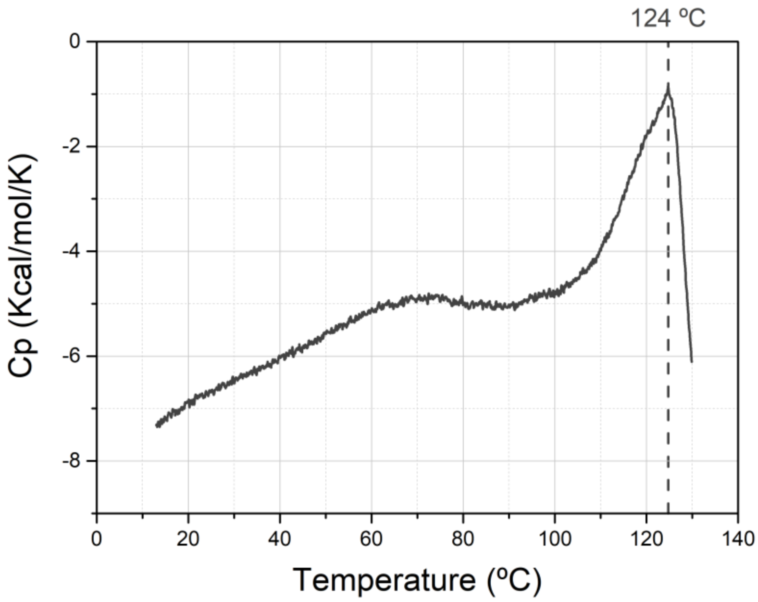

Figure S2. Differential scanning calorimetry thermogram of the thermally induced denaturation of covNHR-SS. The DSC thermogram was recorded at a scan rate of 2°C·min^{-1} at a concentration of 30 μM.

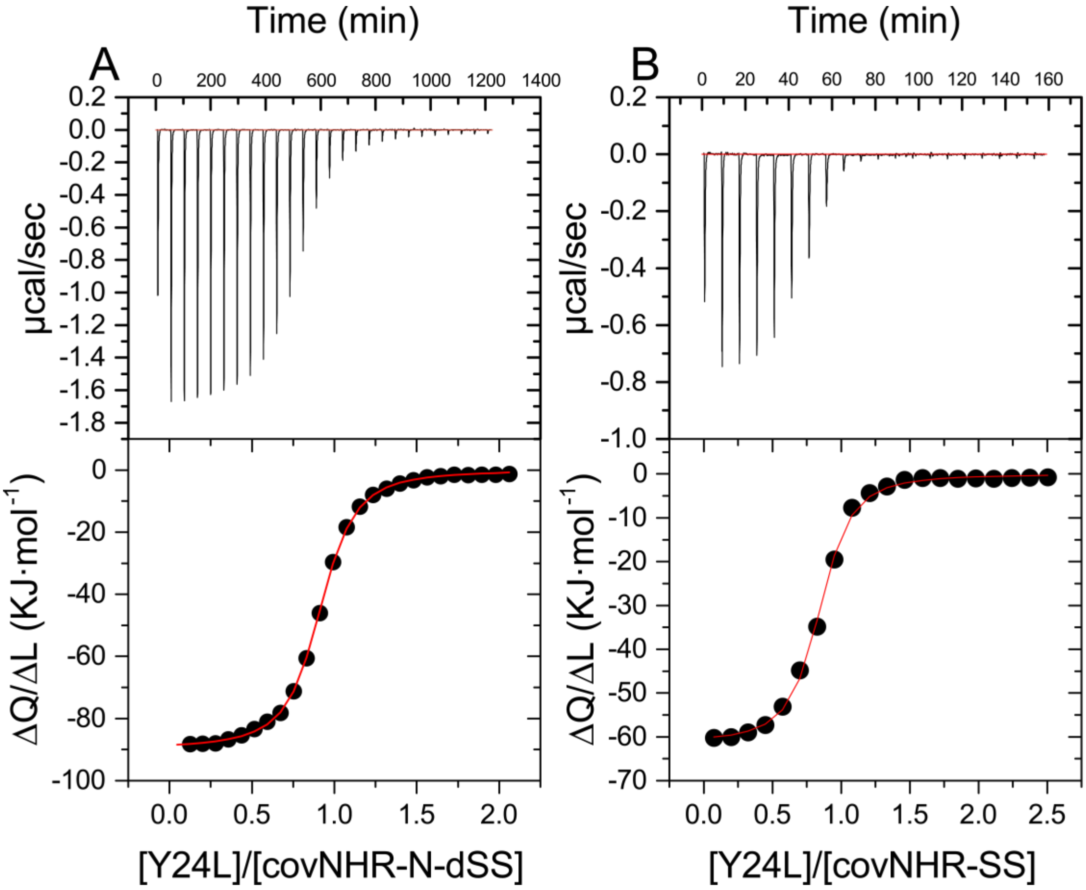

Figure S3. Isothermal titration calorimetry experiments of Y24L peptide binding to the covNHR proteins. **A)** Y24L binding to covNHR-N-dSS and B) to covNHR-SS. The experiments were measured at 25 °C by titration of 10 μM of each miniprotein in the cell with ~300 μM of Y24L peptide from the syringe. The upper panels show the experimental ITC thermograms and the lower panels the normalized binding isotherms. The symbols in the lower panels correspond to the

experimental heats normalized per mole of injected peptide and the lines represent the best fittings using a binding model of n identical and independent sites.

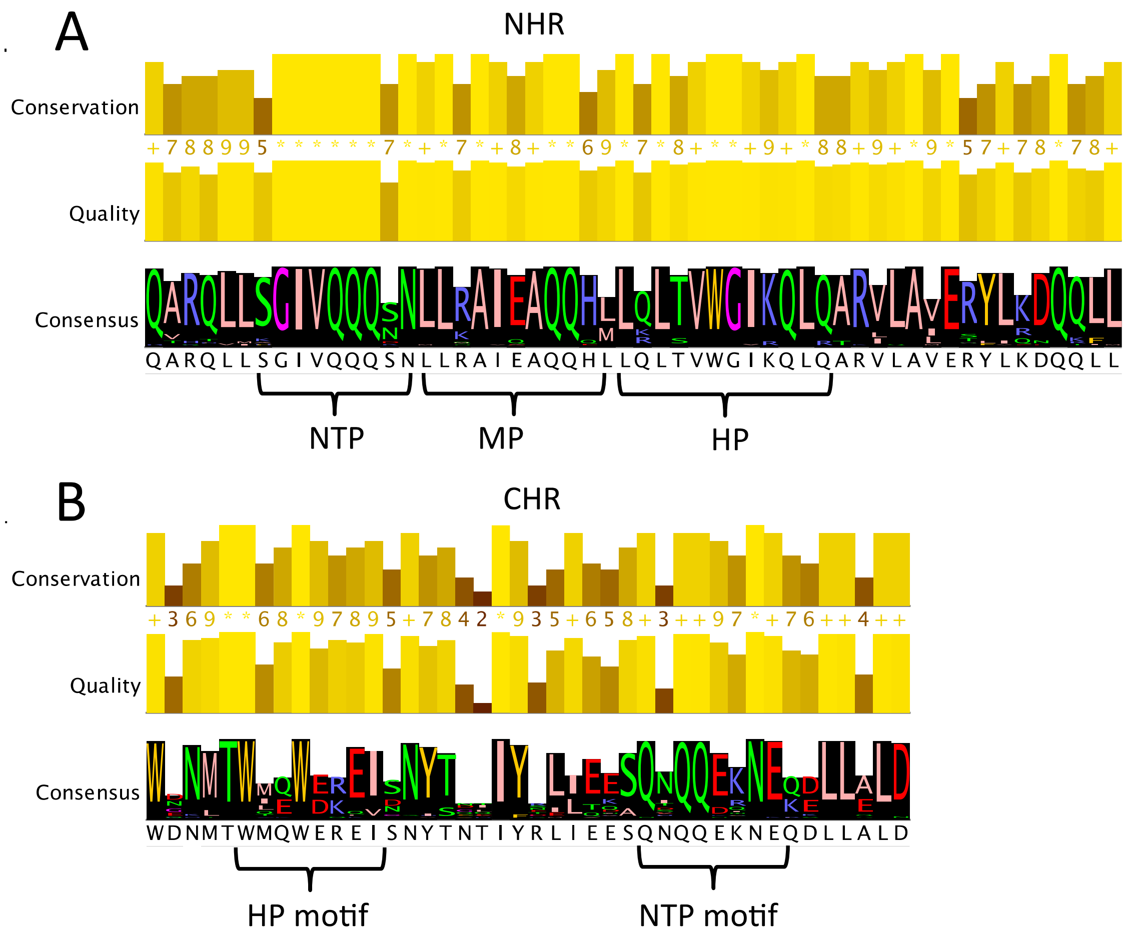

Figure S4. Sequence conservation and consensus sequence of NHR and CHR in gp41. The plots have been made with Jalview [6] using the 2018 Compendium sequence alignment [7] from Los Alamos Sequence Database (https://www.hiv.lanl.gov).

References:

1. Chakrabarti, B. K.; Feng, Y.; Sharma, S. K.; McKee, K.; Karlsson Hedestam, G. B.; LaBranche, C. C.; Montefiori, D. C.; Mascola, J. R.; Wyatt, R. T. Robust Neutralizing Antibodies Elicited by HIV-1 JRFL Envelope Glycoprotein Trimers in Nonhuman Primates. *J. Virol.* **2013**, *87*, 13239–13251.
2. Pialoux, G.; Excler, J. L.; Rivière, Y.; Gonzalez-Canali, G.; Feuillie, V.; Coulaud, P.; Gluckman, J. C.; Matthews, T. J.; Meignier, B.; Kieny, M. P.; et al. A Prime-Boost Approach to HIV Preventive Vaccine Using a Recombinant Canarypox Virus Expressing Glycoprotein 160 (MN) followed by a Recombinant Glycoprotein 160 (MN/LAI). *AIDS Res. Hum. Retroviruses* **1995**, *11*, 373–381.
3. Rerks-Ngarm, S.; Pitisuttithum, P.; Excler, J. L.; Nitayaphan, S.; Kaewkungwal, J.; Premsri, N.; Kunasol, P.; Karasavvas, N.; Schuetz, A.; Ngauy, V.; et al. Randomized, double-blind evaluation of late boost strategies for HIV-uninfected vaccine recipients in the RV144 HIV vaccine efficacy trial. *J. Infect. Dis.* **2017**, *215*, 1255–1263.
4. Zurawski, G.; Shen, X.; Zurawski, S.; Tomaras, G. D.; Montefiori, D. C.; Roederer, M.; Ferrari, G.; Lacabaratz, C.; Klucar, P.; Wang, Z.; et al. Superiority in Rhesus Macaques of Targeting HIV-1 Env gp140 to CD40 versus LOX-1 in Combination with Replication-Competent NYVAC-KC for Induction of Env-Specific Antibody and T Cell Responses. *J. Virol.* **2017**, *91*, 1–20.
5. Cranage, M. P.; Fraser, C. A.; Cope, A.; McKay, P. F.; Seaman, M. S.; Cole, T.; Mahmoud, A. N.; Hall, J.; Giles, E.; Voss, G.; et al. Antibody responses after intravaginal immunisation with trimeric HIV-1CN54 clade C gp140 in Carbopol gel are augmented by systemic priming or boosting with an adjuvanted formulation. *Vaccine* **2011**, *29*, 1421–1430.
6. Waterhouse, A. M.; Procter, J. B.; Martin, D. M. A.; Clamp, M.; Barton, G. J. Jalview Version 2-A multiple sequence alignment editor and analysis workbench. *Bioinformatics* **2009**, *25*, 1189–1191.

7. HIV Sequence Compendium 2018. Brian Foley, Thomas Leitner, Cristian Apetrei, Beatrice Hahn, Ilene Mizrachi, James Mullins, Andrew Rambaut, Steven Wolinsky, and Bette Korber editors. 2018. Publisher: Los Alamos National Laboratory, Theoretical Biology and Biophysics, Los Alamos, New Mexico. LA-UR-18-25673.
